# The changing landscape of drug clinical trials on cardiometabolic diseases in China, 2009–2021

**DOI:** 10.1186/s13098-023-01043-8

**Published:** 2023-04-01

**Authors:** Chen Li, Jun Hao, Yitian Zheng, Chuangshi Wang, Jie Yang, Wenyao Wang, Kuo Zhang, Chunli Shao, Wen Hui, Jiancheng Wang, Wei Li, Yi-Da Tang

**Affiliations:** 1 Department of Cardiology, State Key Laboratory of Cardiovascular Disease, Fuwai Hospital, National Center for Cardiovascular Diseases, Peking Union Medical College, Chinese Academy of Medical Sciences, Beijing, China; 2 Medical Research and Biometrics Center, Fuwai Hospital, National Center for Cardiovascular Diseases, Peking Union Medical College, National Clinical Research Center for Cardiovascular Diseases, Chinese Academy of Medical Sciences, Beijing, 100037 China; 3 Department of Cardiology, Institute of Vascular Medicine, Key Laboratory of Molecular Cardiovascular Science, Peking University Third Hospital, Ministry of Education, Beijing, 100191 China; 4 Department of Science and Technology, West China Hospital, Sichuan University, Chengdu, Sichuan China; 5 Beijing Key Laboratory of Molecular Pharmaceutics and New Drug Delivery Systems, State Key Laboratory of Natural and Biomimetic Drugs, School of Pharmaceutical Sciences, Peking University, Beijing, China; 6 Research Unit of Medical Science Research Management/Basic and Clinical Research of Metabolic Cardiovascular Diseases, Chinese Academy of Medical Sciences, Beijing, China

**Keywords:** Cardiometabolic disease, China, Drug clinical trial, Changing landscape

## Abstract

**Background:**

Cardiometabolic disease is a clinical syndrome characterized by multiple metabolic disorders, with atherosclerosis as the core and cardiovascular and cerebrovascular events as the outcome. Drug research and development (R&D) in cardiometabolic diseases has grown rapidly worldwide. However, the development of cardiometabolic drug clinical trials in China remains unclear. This study aims to depict the changing landscape of drug clinical trials for cardiometabolic diseases in China during 2009–2021.

**Methods:**

The detailed information of drug trials on cardiometabolic diseases registered in the National Medical Products Administration (NMPA) Registration and Information Disclosure Platform was collected between January 1, 2009, and July 1, 2021. The landscape of cardiometabolic drug clinical trials was analyzed by the characteristics, time trends, indications, pharmacological mechanisms, and geographical distribution.

**Results:**

A total of 2466 drug clinical trials on cardiometabolic diseases were extracted and analyzed. The annual number of drug trials increased rapidly in the past twelve years. Among all the trials, the bioequivalence trials (1428; 58.3%) accounted for the largest proportion, followed by phase I (555; 22.5%), phase III (278; 11.3%), phase II (169; 6.9%), and phase IV (26; 1.1%). Of 2466 trials, 2133 (86.5%) trials were monomer drugs, only 236 (9.6%) trials were polypills and 97 (3.9%) were traditional Chinese medicine (TCM) compounds. In terms of pharmacological mechanisms, the number of trials in dihydropyridine (DHP) calcium antagonists 321 (11.9%) ranked first, while trials in angiotensin receptor blocker (ARB) 289 (10.7%) and dipeptidyl peptidase-4 (DPP-4) inhibitor 205 (7.6%) ranked second and third place respectively. Of 236 chemical polypills trials, 23 (9.7%) polypills were the combination of DHP calcium antagonists and statins, while others were the combination of two same pharmacological effect agents. As for the geographical distribution of leading units, 36 trials were led by principal investigators (PI) units from Beijing, followed by Jiangsu (n = 29), Shanghai (n = 19), Guangdong (n = 19), and Hunan (n = 19), showing an uneven regional distribution.

**Conclusions:**

Great progress has been made in drug clinical trials on cardiometabolic diseases, especially in antihypertensive agents, hypoglycemic agents, and hypolipidemic agents. However, the insufficient innovation of first-in-class drugs and polypills should be carefully considered by all stakeholders in drug trials.

**Supplementary Information:**

The online version contains supplementary material available at 10.1186/s13098-023-01043-8.

## Introduction

The incidence of cardiovascular diseases (CVDs) was approximately 330 million and was on the rise, and the mortality rate remained the highest among all diseases in China [1]. Since metabolic syndrome and cardiometabolic risk have been formalized, the risk of CVD attributable to metabolic factors has been gradually acknowledged [2]. Cardiometabolic disease is a clinical syndrome characterized by multiple metabolic disorders, with atherosclerosis as the core and cardiovascular and cerebrovascular events as the outcome. The concept of cardiometabolic disease was proposed and widely recognized by the medical community [3]. Several studies confirmed that cardiometabolic diseases have shown a prevalent trend in China and worldwide [4–6]. The broad concept generally refers to all CVDs related to metabolic disorders. In this study, we mainly focus on the six most closely related diseases, including coronary heart disease, hypertension, type 2 diabetes, obesity, dyslipidemia, and stroke [4]. Owing to the overlapping of pathological mechanisms, drugs targeting cardiometabolic disease usually shared similar targets and exhibited definite cardio-protection effects. The development of cardiometabolic drugs and the progress of their clinical trials will help promote the comprehensive treatment and management of cardiometabolic diseases.

In China, there are three common registration platforms for clinical trials, namely ClinicalTrials.gov, WHO International Clinical Trials Registry Platform, and Chinese Clinical Trials Registry (ChiCTR), the registration on these platforms is not mandated by the supervision department. The National Medical Products Administration (NMPA) Registration and Information Disclosure Platform for Drug Clinical Studies was established in 2013 and all drug trials approved by the NMPA must be registered and disclosed on the platform. Meantime, a series of policies were also issued by the Chinese government to improve drug research and development (R&D). The review and approval process of drug clinical trials was accelerated since then. Analysis of the platform data could also reflect the actual situation of drug trials in China [7]. Several studies analyzed drug trials on lung cancer, liver diseases, lymphoma, etc [8–11]. This study aimed to provide insight into the changing landscape in the drug clinical trials for cardiometabolic diseases in China, to identify unmet clinical needs and to provide supportive data for investigators, doctors, pharmaceutical enterprises, policymakers, and other stakeholders.

## Methods

### Data source

The data of drug clinical trials on cardiometabolic diseases were acquired through the NMPA Registration and Information Disclosure Platform for Drug Clinical Studies (http://www.chinadrugtrials.org.cn). The platform was established by the NMPA in 2013. To unify supervision, the NMPA required that all registered drug clinical trials must be compulsorily registered on this platform, and supplementary registration must be completed within 3 months for clinical trials of drugs before 2013. Therefore, the NMPA registration platform is representative and authoritative in drug clinical trials in China. The registration items of clinical trials are available publicly on the platform.

### Search strategy and selection criteria

**Fig. 1:**
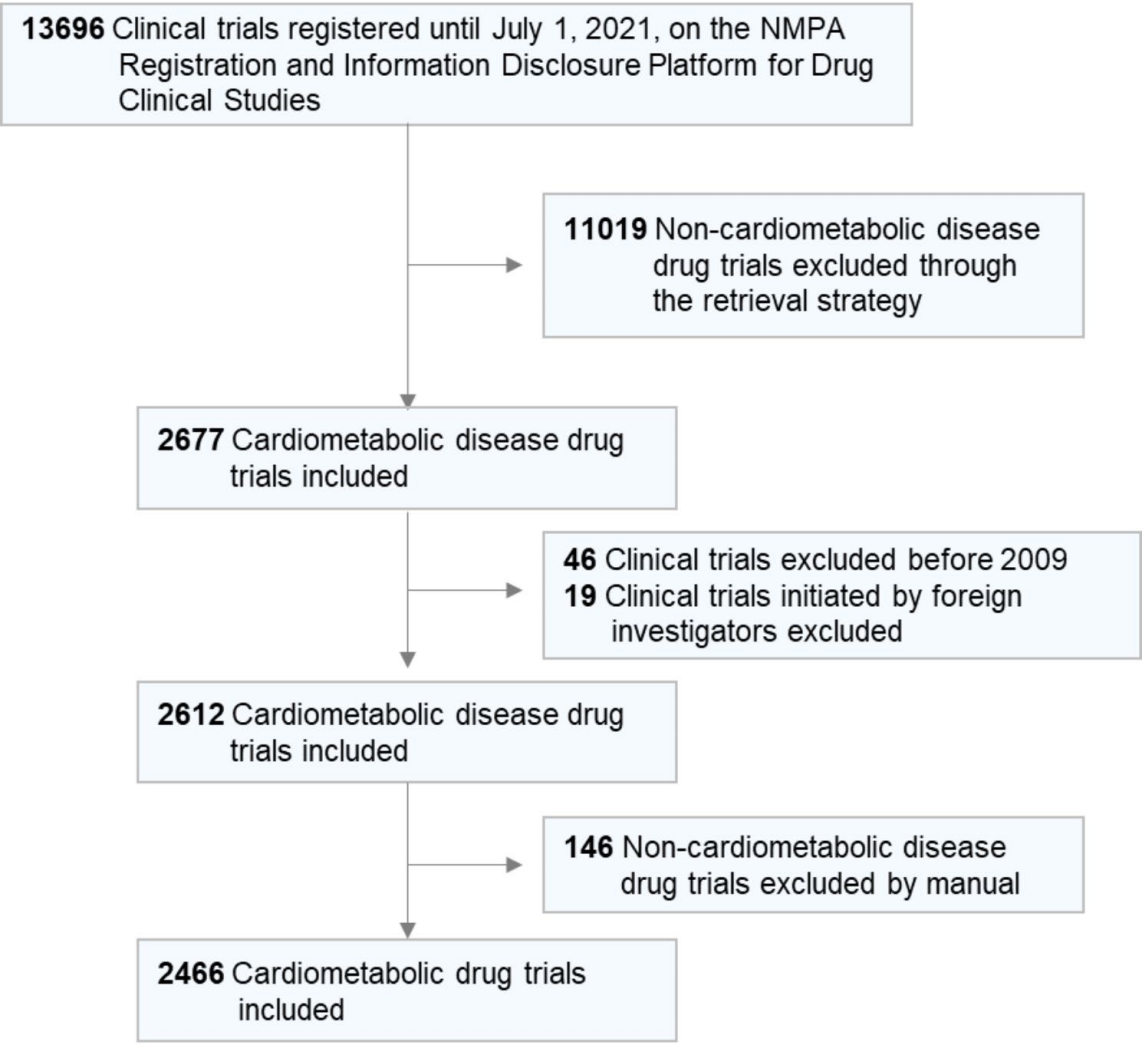
**Flowchart of the selection process** Abbreviations: NMPA, National Medical Products Administration

To fully cover the drug trials of the cardiometabolic diseases category, we used “coronary heart disease”, “stroke”, “type 2 diabetes”, “hypertension”, “dyslipidemia”, and “obesity” as key indications for independent search. The search strategy was established as comprehensive as possible by three cardiologists (C.L., Y.T.Z. and W.Y.W.). Details of search terms were provided in the Supplemental Materials. In 2009, a program launched by China’s Ministry of Science and Technology aimed to support innovative drug R&D, boosting the clinical trials for cardiometabolic drugs in China. Therefore, the starting and ending dates were January 1, 2009, and July 1, 2021, respectively. The included drug clinical trials must meet the following criteria. Firstly, the clinical indication of the experiment must be one of the cardiometabolic diseases: coronary heart disease, stroke, type 2 diabetes, hypertension, dyslipidemia, and obesity. Secondly, the experiment was mainly conducted in China. Detailed information was collected for further analysis: trial title, study design, study type, the phase of trials (I–IV, other), indications, trial objective, drug type and mechanism of action, date of submission, geographic region of the leading unit and participating centers, study locations, number of participating centers, recruitment status, inclusion and exclusion criteria. Two investigators (C.L. and J.H.) reviewed the information of trials independently. In case of disagreement, all investigators discussed and reached a consensus finally. After carefully screening, 2466 trials were included in the final analysis (Fig. 1).

### Data analysis and statistical analysis

According to the drug type, trials were divided into four categories: chemical drug, biological product, Chinese herbal monomer (natural product), and traditional Chinese medicine (TCM) compound. We also classified the trials into three groups according to the drug composition: monomer drug, chemical polypill, and TCM compound. The changes in characteristics and trends of clinical trials were analyzed from the perspectives of the numbers, as well as the distribution of indications, drug mechanisms, and geographical locations. The characteristic of clinical trials was described with numbers and percentages (%). All statistical analyses were performed with SAS 9.4 (SAS Institute, Cary, NC, USA).

## Results

### Time trends of registered drug clinical trials

**Fig. 2:**
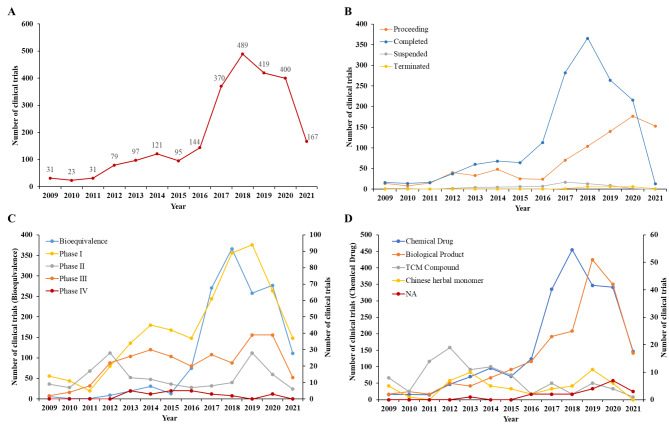
**Annual numbers of initiated cardiometabolic drug clinical trials by major indications in China, 2009–2021** The data ends in July 1st, 2021

2466 drug clinical trials for cardiometabolic diseases were registered on the platform from January 1st, 2009 to July 1st, 2021. During 2009–2013, The annual number of trials registered each year was quite low with a slow increase, while the number of registered trials grew rapidly from 2013 to 2020, presented a 2.5-fold change in 2017, and reached a peak at 489 in 2018 (Fig. 2A). Consistent with the trend of registered number, the change in the annual number of proceeding and completed trials showed the similar trend, while the number of terminated or suspended trials remained at a low level of less than 20 all the time (Fig. 2B). Among the five trial phases, the bioequivalence study accounted for more than the half proportion (58.3% [1428/2466]), followed by phase I (22.5% [555/2466]) and phase III (11.3% [278/2466]). The phase II and phase IV only represented 6.9% and 1.1% respectively (Table 1; Fig. 2C). Unlike the steady growth of other phases, the number of bioequivalence studies has exceeded that of phase I since 2015 and presented a dramatic increase between 2015 and 2020. The trials were reclassified into chemical drug, biological product, Chinese herbal monomer, and TCM compound. The chemical drugs accounted for the most proportion (84.3% [2079/2466]) and the biological products took the second place with 209 trials (8.5%) (Table 1). During 2009–2021, the two major types increased steadily, while the number of Chinese herbal monomers and TCM compounds rosed from 2009 to 2013 and decreased gradually (Fig. 2D).

**Table 1:** Characteristics of Cardiometabolic Drug Clinical Trials in China

Classification	Frequency(%)
Status	
Proceeding	851 (34.5%)
Completed	1529 (62.0%)
Suspended	20 (0.8%)
Terminated	66 (2.7%)
**Trials Phase**	
Phase IV	26 (1.1%)
Phase III	278 (11.3%)
Phase II	169 (6.9%)
Phase I	555 (22.5%)
Bioequivalence	1438 (58.3%)
**Type of drugs**	
Chemical Drug	2079 (84.3%)
Biological Product	209 (8.5%)
TCM Compound	97 (3.9%)
Ingredient of Natural Drug	60 (2.4%)
NA	21 (0.9%)
**Design**	
Single-arm	100 (4.1%)
Crossover	1663 (67.4%)
Parallel-group	698 (28.3%)
Factorial design	5 (0.2%)
**Randomization**	
Randomization	2353 (95.4%)
Non-randomization	113 (4.6%)
**Blinding**	
Open-label	1867 (75.7%)
Single-blind	35 (1.4%)
Double-blind	564 (22.9%)
**Coverage**	
Domestic	2401 (97.4%)
International Multi-center	65 (2.6%)

Abbreviations: NA. Not applicable

### Characteristics of registered drug clinical trials

In terms of the study design, the crossover study accounted for the largest proportion (67.4% [1663/2466]), followed by the parallel-group study (28.3% [698/2466]) and (4.1% [100/2466]) in single-arm and factorial design (0.2% [5/2466]). Among all the trials, the randomization study (95.4% [2353/2466]) was in the majority compared with the non-randomization study (4.6% [113/2466]). Regarding the blind design, the open-label trials were 1867 (75.7%), while the double-blind and single-blind trials were only 564 (22.9%) and 35 (1.4%) respectively. In addition, the domestic trials accounted for 97.4% compared to trials covering international multi-center 2.6% (Table 1). We further analyzed the distribution of phases and the status of the trials. The suspended and terminated trials in phase III (7.9% [22/278]) accounted for the majority, and 3.4% [19/555] in phase I and 3.0% [43/1438] in the bioequivalence study (Supplemental Fig. 1).

### Distribution of clinical trials by drug types and mechanisms in China

**Fig. 3:**
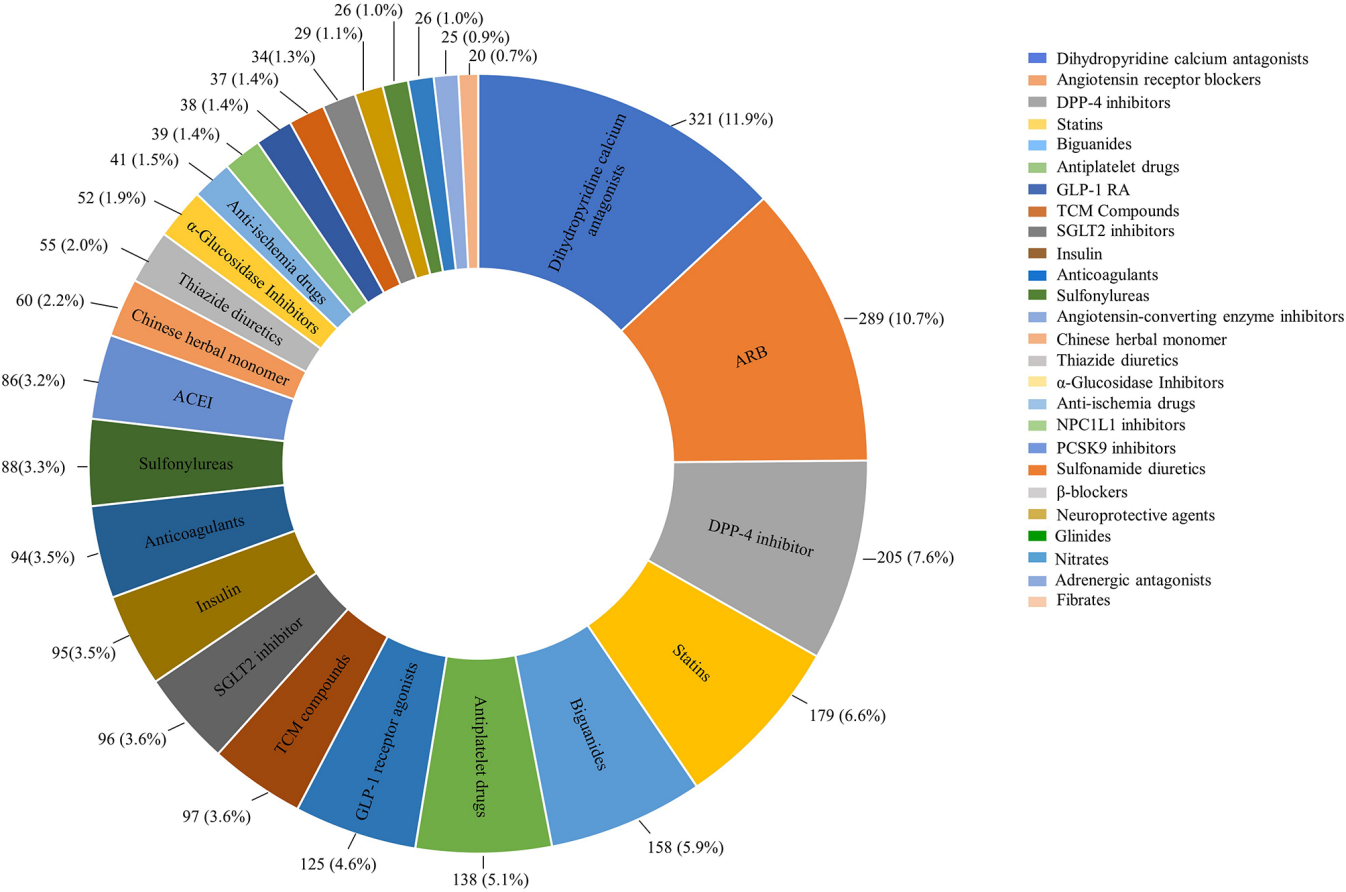
**Distribution of clinical trials by drug types and mechanisms** The total number of clinical trials would be more than 2466, as a clinical trial could have more than one drug type. Abbreviations: DDP-4, dipeptidyl peptidase-4; GLP-1 RA, glucagon-like peptide-1 receptor agonists; SGLT2, sodium-dependent glucose transporters 2; NPC1L1, Niemann-Pick C1 Like1; PCSK9, Proprotein Convertase Subtilisin/Kexin Type 9

Drug trials were further analyzed according to the pharmacological mechanisms. Drug trials with a registered number over 20 were summarized in Fig. 3 and the others were detailly summarized in Supplemental Table 1. Among all the drug trials, the number of trials in dihydropyridine (DHP) calcium antagonists 321 (11.9%) ranked first, while trials of angiotensin receptor blocker (ARB) 289 (10.7%) and dipeptidyl peptidase-4 (DPP-4) inhibitor 205 (7.6%) took the second and third place (Fig. 3). The top ten registered drugs also included statins 179 (6.6%), biguanides 158 (5.9%), antiplatelet drugs 138 (5.1%), glucagon-like peptide-1 (GLP-1) receptor agonists 125 (4.6%), TCM compounds 97 (3.6%), sodium-dependent glucose transporters 2 (SGLT2) inhibitor 96 (3.6%) and insulin 95 (3.5%). Chinese herbal monomers also accounted for 2.2% (60) of the cardiometabolic drug trials while Ginkgolides (23.3% [14/60]) and Ginsenoside (23.3% [14/60]) had the majority in the type of drugs. Besides, the registered number of inhibitors targeting Niemann-Pick C1 Like1 (NPC1L1) 39 (1.4%) and Proprotein Convertase Subtilisin/Kexin Type 9 (PCSK9) 38 (1.4%) also took a place in the top twenty. Additionally, other types of new drug trials were less than ten, including mesenchymal stem cell, angiotensin receptor-neprilysin inhibitor (ARNI), G protein-coupled receptor (GPR40) agonist, islet amyloid polypeptide (IAPP) analog, and hepatocyte growth factor.

### Indications of registered clinical trials

**Fig. 4:**
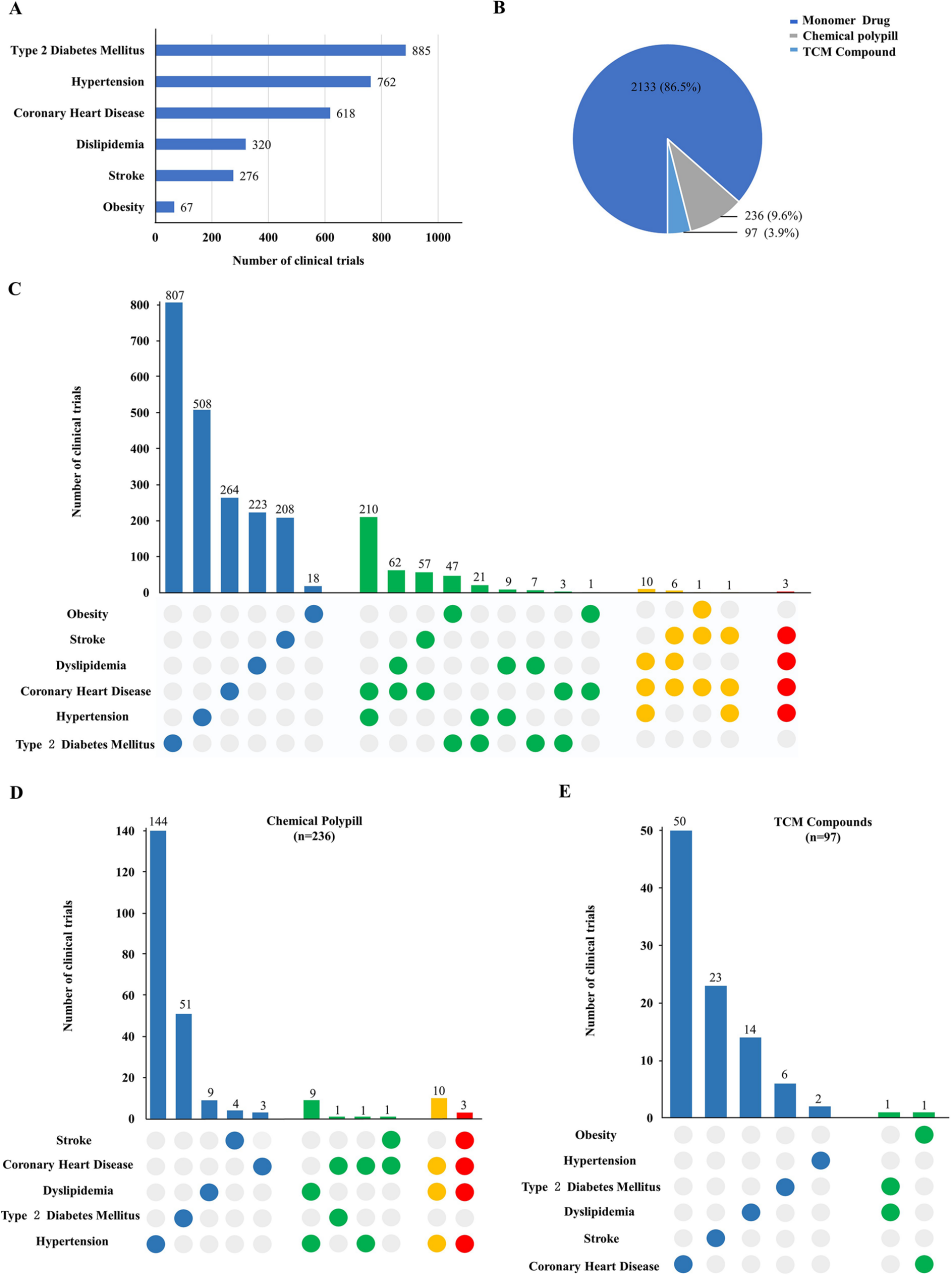
**Distribution of indications in cardiometabolic drug clinical trials** (A) Number of clinical trials according to the indication in cardiometabolic diseases. (B) Trials are divided into monomer drug, chemical polypill, and TCM compound. (C) The number of drug trials for different indications. Blue represents one indication. Green represents two indications. Yellow represents three indications, and red represents four indications. (D) In the chemical polypill, the number of drug trials for different indications. The blue represents one indication which suggested that the polypill for one indication. The green represents two indications for polypill. The yellow represents three indications and the red represents four indications. (E) In TCM compounds, the number of drug trials for different indications. The blue represents one indication and the green represents two indications. Numbers above each column of the chart represent the actual number of trials. Abbreviations: TCM, Traditional Chinese Medicine

The clinical indication coverage of drug trials was illustrated in Fig. 4A according to the cardiometabolic disease categories. The top three indications were Type 2 diabetes, hypertension, and coronary heart disease with the number of 885, 762, and 618 respectively, while there were 320 trials for dyslipidemia, 276 for stroke, and 67 for obesity. It is worth noting that the drug may cover one or more indications and the indications may overlap. The mono-indication drug trials had the major proportion, while the number of trials covering two indications accounted for the largest proportion (417 [95.2%]) of poly-indication drugs (n = 438). Among these, drugs for hypertension and coronary heart diseases had the largest number (210 [50.3%]), followed by dyslipidemia and coronary heart diseases (62 [14.8%]) (Fig. 4C). The underlying drug types in the two poly-indication categories were almost dihydropyridine (DHP) calcium antagonists and statins. In addition, the trials cover three and four indications in the poly-indication drugs were almost polypills. We classified the drug trials into monomer drugs, chemical polypills, and TCM compounds according to the ingredients of drugs. Monomer drugs accounted for the highest proportion (86.5% [2133/2466]), while the chemical polypills and TCM compounds only accounted for a low proportion (9.6% [236/2466]) and (3.9% [97/2466]) respectively (Fig. 4B). The therapeutic effect of polypill on CVD has been confirmed [12, 13]. In terms of the chemical polypill, there are not only drug combinations of the same pharmacological effects for optimal therapy in single indications, but also drug combinations of different pharmacological effects for one indication or multiple indications [14]. The polypills for hypertension had the largest proportion (61.0% [144/236]) followed by type 2 diabetes (21.6% [51/236]) and dyslipidemia (3.8% [9/236]). Figure 4D showed the polypill for two and more indications. The number of polypills for hypertension and dyslipidemia was 9, 10 for the above two indications with coronary heart disease added, and 3 for the combination of the above three indications with stroke (Fig. 4D). The polypills in these three types were all a combination of DHP calcium antagonists and statins. In contrast to the chemical polypill, the indications of TCM compound mainly focused on coronary heart disease (51.5% [50/97]) and stroke (23.7% [23/97]) respectively (Fig. 4E).

**Fig. 5:**
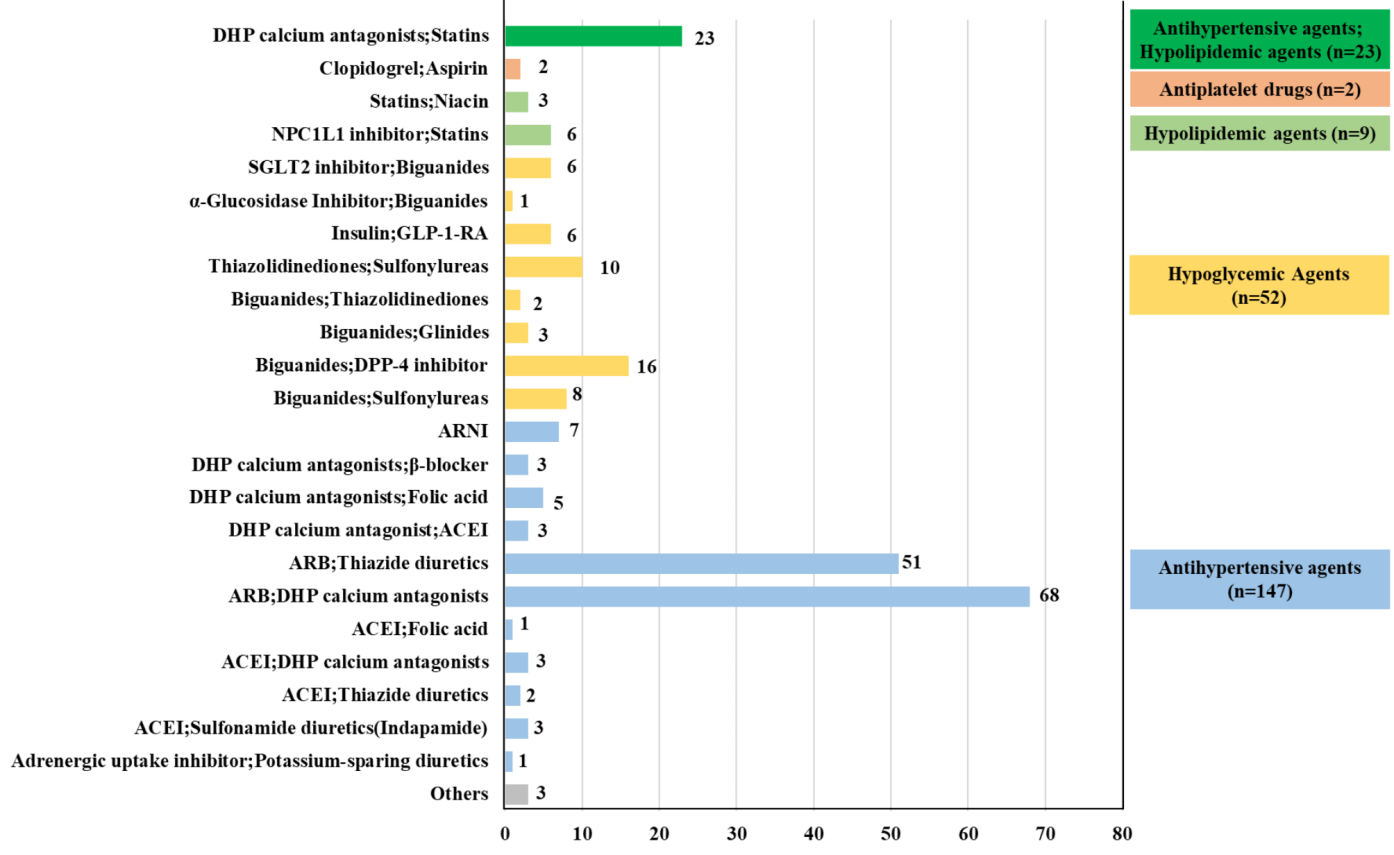
**Distribution of clinical trials by drug mechanism in chemical polypills** Numbers beside each column of the chart represent the actual number of trials. Abbreviations: DHP, dihydropyridine; SGLT2, sodium-dependent glucose transporters 2; NPC1L1, Niemann-Pick C1 Like1; ACEI, angiotensin-converting enzyme inhibitor; ARB, angiotensin receptor blocker; DDP-4, dipeptidyl peptidase-4; ARNI, angiotensin receptor-neprilysin inhibitor; GLP-1 RA, glucagon-like peptide-1 receptor agonists

Figure 5 illustrated the distribution of clinical trials by drug mechanism in chemical polypills. From the perspective of drug ingredients in chemical polypills, the majority type was the combination of two drugs with the same pharmacological effect, including antihypertensive agents combination (n = 147), hypoglycemic agents combination (n = 52), hypolipidemic agents combination (n = 9), and antiplatelet drugs combination (n = 2). These polypills offered a better choice to optimize the management of hypertension, dyslipidemia, and diabetes. However, there was only one type of drug combination with two different pharmacological effects including statins and DHP calcium antagonists, which reflected the lack of polypills in multi-indication therapy in cardiometabolic diseases.

### Geographical distribution of clinical trials according to leading units

**Fig. 6:**
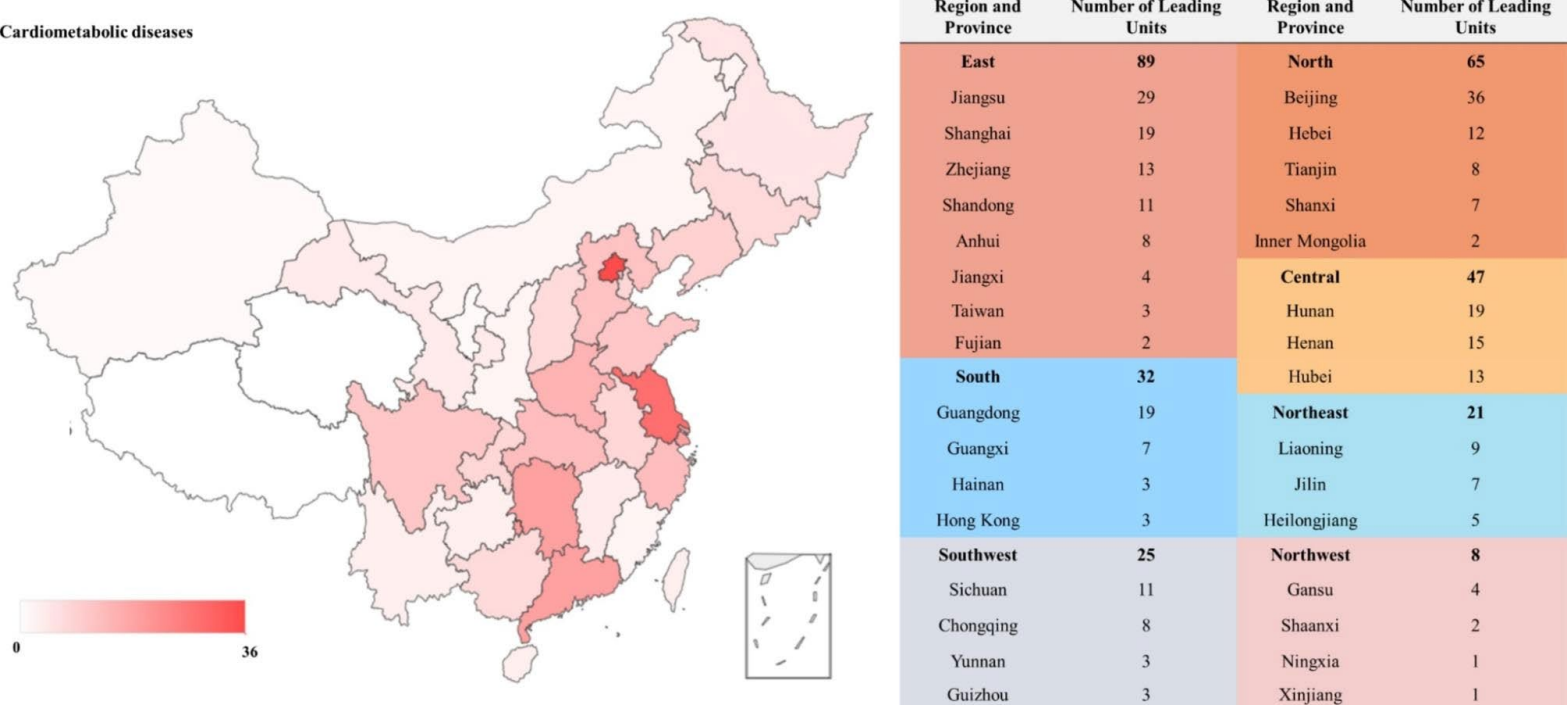
Geographical distribution of leading units in China

The 2466 drug trials for cardiometabolic diseases were conducted in 31 provinces and regions across China (Fig. 6). The east of China had the largest number of principal investigators (PI) leading units (n = 89), followed by the north of China (n = 65). The PI leading units from northwest China were quite few (n = 8). In terms of certain provinces, the number of PI leading units from Beijing (n = 36) ranked first, followed by Jiangsu (n = 29), Shanghai (n = 19), Guangdong (n = 19), and Hunan (n = 19). By contrast, there was only one PI leading unit from Xinjiang and Ningxia respectively. The geographical distribution of cardiometabolic drug trials was severely uneven. The detailed geographical distribution of PI leading units according to the six indications in cardiometabolic diseases was provided in Supplemental Fig. 2, which also showed the same trend in Fig. 6.

## Discussion

From the famous Framingham Heart Study in the last century, it was found that CVDs were attributable to metabolic factors [15, 16]. The concepts of metabolic syndrome and coronary heart disease risk equivalents promoted the acknowledgment of metabolic disorders in the pathological development of CVDs by the medical community [4]. Epidemiological studies have confirmed that the morbidity of adverse cardiovascular events significantly increased in patients with type 2 diabetes, hyperlipidemia, hypertension, or obesity [17–21]. The core pathological mechanisms of these metabolic disorders are insulin resistance and chronic inflammatory signal activation, which involve a variety of mechanisms and signals and form a complex regulatory network [22–24]. More importantly, these metabolic diseases bring pathological effects to the cardiovascular system directly or indirectly. These metabolic disorders often co-existed and have reciprocal causation, which induced atherosclerosis and finally resulted in major adverse cardiac and cerebrovascular events (MACCE) [25, 26]. Thus, it is important to take all kinds of metabolic factors into comprehensive management, prevention, and intervention for patients with cardiometabolic diseases [3]. Also, the newly discovered drugs for diabetes and dyslipidemia have been confirmed with definite cardiovascular benefits, which also suggested that the drug therapy of cardiometabolic diseases should be considered as a whole instead of separate diseases. Regarding the clinical trials of drugs in China, several relevant analyses and evaluations have been conducted in the fields of lung cancer, endocrine disorder, liver disease, etc [8, 11, 27, 28]. However, the clinical trials of cardiometabolic diseases in China and their changes have not been fully concerned and discussed. To the best of our knowledge, this is the first study to depict and analyze the changing landscape of drug clinical trials on cardiometabolic diseases in China. Our analysis is helpful to understand the progress and change trend of drug trials on cardiometabolic diseases and provides evidence support for future drug discovery and clinical strategy.

We analyzed 2466 trials on cardiometabolic diseases from 2009 to 2021 in China. Due to policy promotion and a large amount of investment, the cardiometabolic drug trials have made great progress in the past 12 years. Since 2015, a series of documents issued by the State Council of China has made the approval process of new drug trials significantly standardized and accelerated after several reforms of the approval system, including “Opinions on Reforming the Evaluation and Approval System of Pharmaceutical and Medical Devices” in 2015 and “ Opinions on Deepening the Reform of the Evaluation and Approval System and Encouraging the Innovation of Pharmaceutical and Medical Devices” in 2017 [29, 30]. It could also be verified by the rapidly increased registered number of cardiometabolic trials since 2015 in Fig. 2. The NMPA has also adopted an implied licensing system for clinical trials of new drugs. If the application was not rejected or questioned within 60 days after application, pharmaceutical enterprises could carry out clinical trials as planned [31]. The efficacy and safety of drugs were finally evaluated by real clinical data and no strict review would be set at the entrance of trial applications, which further accelerated the drug trials in China. In addition, the bioequivalence study could not be ignored in the increase of clinical trials. To encourage drug clinical trials and popularize drug use, the “Opinions on Carrying out the Evaluation of the Consistency of the Quality and Efficacy of Generic Drugs” was issued by the State Council in 2016 [32]. Since then, a high proportion of generic drugs and bioequivalence trials has become the mainstream of applications. In our results, although the number of phase I trials was still rising in cardiometabolic disease, from 2016 onwards, the proportion of bioequivalence studies exceeded that of phase I trials (Fig. 2C) and reached nearly three times of phase I trials in total (1438/555). Although the huge growth of bioequivalence studies provides convenience for clinical medication, it also reflects the insufficient innovation ability in first-in-class drug research, which requires long-term investment and effort. Besides, the percentage of double-blind trials (22.9%) were relatively low. Also, the registered number of international multi-center trials was only a few (65; 2.6%), which might limit the utilization of new drugs in Chinese patients.

It is also worth noting that due to the standardized and stricter supervision than investigators-initiated trials, the number of TCM compound trials for the market showed a downward trend since 2013 (Fig. 2D), which might promote the healthy development of TCM medicine in cardiometabolic fields. Due to the complex ingredients of compounds and unclear pharmacological effects in TCM, despite clinical practice verification, the lack of high-quality randomized controlled trials (RCT) limited its worldwide promotion. Several TCMs’ RCTs achieved positive results in CVDs in recent years, which demonstrated the huge potential of TCM in the treatment of CVDs [33–35]. The development of drug clinical trials on Chinese herbal monomers (natural products) and TCM compounds will bring great benefits to cardiometabolic disease therapy.

As the cardiometabolic disease is a clinical syndrome with multiple co-existed diseases, in terms of cardiometabolic drug therapy, the poly-indication drug trials help to improve the drug use efficiency for clinical therapy and reduce the number of drugs for patients. In terms of the indications’ coverage, the drugs covering single indications account for the majority, of which hypoglycemic drugs and antihypertensive drugs made up a high proportion, while trials covering multiple indications are much less. This result indicates that there is still potential improvement in expanding the indications of cardiometabolic drugs in clinical trials. More importantly, the drug discovery on CVD needs to be considered as a whole from bench to bedside. Targets in the insulin resistance and inflammatory signaling network will still be the “gold mine” for cardiometabolic new drug discovery. What’s more, with the development of network pharmacology, natural products, and TCM compounds also have great potential for future discovery [36].

Compared with a single drug, drug combination therapy could improve efficacy, reduce dosage and side effects, cut down the cost of drug use, and improve the primary and secondary prevention effects of cardiometabolic diseases. Several trials have proved that the polypill containing antihypertensive drugs and statins or aspirin as an addition could significantly increase the efficiency in the prevention of cardiometabolic diseases [12, 37−40]. Development of polypills has great value in reducing cardiovascular risks worldwide. Especially in developing countries, the use of polypills could also significantly reduce the economic burden on patients and medical security funds while improving the prevention and treatment effects [13]. In terms of cardiometabolic disease, there is one more benefit of polypills for the overlap of pathological mechanisms of diseases and the underlying multi-therapeutic effects. In our results, we found that among the polypills, the majority was the combination of two same mechanisms in the control of hypertension, diabetes, or dyslipidemia. However, the drug combination of different pharmacological mechanisms was only the polypill containing DHP and statins, lacking other combinations including but not limited to proven effective antihypertensive drugs plus statins and aspirin. It is indicated that the development of polypills in cardiometabolic diseases needs further investment and research. However, the combinatorial strategy was not always successful. Pharmaceutical enterprises should also consider potential risks in developing such polypills. For instance, the ILLUMINATE trial (NCT00134264) of torcetrapib/atorvastatin combination unexpectedly increased the mortality. And the angiotensin-converting enzyme inhibitors (ACEIs) and angiotensin receptor blockers (ARBs) combination failed because of the increased risks of hyperkalemia, and renal insufficiency [41, 42]

From the perspective of drug mechanism, the top 20 drug trials were dominated by antihypertensive drugs, hypoglycemic drugs, and lipid-lowering drugs. Among various classic drugs, many clinical trials of DHP calcium antagonists, statins, insulin, antiplatelet drugs, ACEIs, and ARBs provided more choices for patients and indirectly reflected the extensive demand in clinical application. In terms of trials on new drug targets, multiple studies including the LEADER study, the HARMONY study, and the EMPEROR-Preserved study have proved that GLP1-RA and SGLT2 showed obvious cardiovascular benefits [43–47]. The FOURIER study and ODYSSEY study have also established the PCSK9 antibody’s position in the therapy of hypercholesterolemia and prevention of atherosclerotic coronary artery disease (ASCVD) and stroke [48–50]. For the above hot areas, the number of applications for cardiometabolic drug trials accounted for a large proportion and many of them belonged to Class I drugs, which indicated that Chinese drug enterprises have a certain ability in the fast-follow drugs and have made noticeable progress in drug discovery. The rapid growth of investment in hot target drug discovery may provide more choices for patients’ clinical medication. However, it should not be ignored that the R&D of hot drugs may lead to intense homogenization competition and waste of resources. Besides, it is harmful to differential development either. The lack of first-in-class drugs and excessive competition around a few drug targets indicate that the ability of innovative cardiometabolic drug discovery is still weak. The Chinese pharmaceutical enterprises have to choose targets that have been verified by international pharmaceutical enterprises to do minimal innovation and fast follow.

In the 1980s, a large number of drugs, especially statins, were approved by the FDA for marketing, leading to the rapid development of cardiovascular drugs [51]. Compared with the rapid development of drug discovery and trials in cancer, drug R&D in the cardiovascular field is relatively slow, [52, 53]. which is partly due to the widely recognized efficacy and safety of approved drugs. More importantly, there are many limitations on cardiometabolic drug R&D including the complex mechanism of CVD, more conservative recommendations in the related guidelines, and stricter requirements for superiority evaluation and safety. Thus, many enterprises reduced their investment and even quit the cardiovascular field for the sake of high risk without profits. It is imperative to further optimize the process of cardiometabolic clinical trials and consider new paths to reduce costs. The most powerful strength to change the current situation comes from the clinical demands. On the one hand, it is necessary to adhere to the investment in research from basic to clinical study on cardiometabolic drug discovery. Basic researchers and clinical trial stakeholders should closely cooperate in the process from bench to bedside. On the other hand, another effective strategy is to expand the indications for existing approved drugs. Besides, precision medicine and individual medicine may help to find more precise targets to better focus the target patients. For instance, the detection of CYP2C19 variant alleles could guide individualized antiplatelet therapy, which is beneficial to the precise strategy and reduce the scale of the trial and costs as well. RNAi-targeted therapy also shows broad prospects in dyslipidemia [54, 55]. Inclisiran, a siRNA targeting PCSK9, has already been registered for two trials in China. Also, many new technologies may bring breakthroughs including high-throughput screening platforms (small molecules and natural products), multi-omics technology, artificial intelligence (AI) technology, [56, 57] human induced pluripotent stem cell-derived cardiomyocyte (hiPSC-CM), [58, 59], 3D bioprinting organoid, [60, 61] and the humanized genetic modified models, [62, 63]. which may greatly reduce the cost and risk of new drugs before the clinical trials.

Despite the much progress in the drug clinical trials on cardiometabolic disease, there are still some obvious problems. Firstly, the number of international multi-center trials was quite few. The international multi-center trials might provide Chinese patients with more opportunities to use new drugs and also help promote the new drugs worldwide. Secondly, the lack of original new drugs in this field should not be ignored. Compared with the fierce competition in hot targets like PCSK9 and SGLT2 and “me too” drugs, there were hardly any first-in-class drugs in this field, which may be due to insufficient basic research and the lack of enterprises with abundant capital and long-term experience. Thirdly, we also found that there was a severely uneven geographical distribution in cardiometabolic disease trials according to the PI leading units. The numbers of PI leading units located in the east and north of China were much more than those in the northeast and northwest of China, which indicated that the distribution of clinical trial units was strongly related to the level of economic development. The imbalance should be improved by gradual resource input and construction.

Our study also has some limitations. Firstly, despite the rigorous data processing, there may be potential deviations in manual retrieval and screening. Secondly, the clear name and drug classification of some drug trials were not disclosed in the platform by pharmaceutical enterprises due to commercial confidentiality, which resulted in failure to be analyzed. Finally, because of the availability of data, the scope of our study is limited to China, but further research on cardiometabolic disease drug clinical trials is worth conducting from a global perspective to enlighten the R&D in cardiometabolic disease drugs.

## Conclusions

This study was the first study to provide a comprehensive landscape of cardiometabolic disease drug clinical trials in China in the past 12 years. Owing to the strong demand for clinical application and sustained government policy support, drug clinical trials on cardiometabolic disease have developed rapidly, especially in the field of antihypertensive agents, hypoglycemic agents, and hypolipidemic drugs. It may be necessary to pay more attention to overlapping areas and precise treatment, as well as to the improvement of the delivery system, polypills, and dosage form. To pass the high wall in cardiometabolic drug R&D, more policy support is essential to find a breakthrough. The progress of drug trials on the cardiometabolic disease will not only benefit patients in China but also contribute to the development of global drug R&D.

## Electronic supplementary material

Below is the link to the electronic supplementary material.

Supplementary Material 1

## Data Availability

All data generated or analyzed during this study are included in this published article.

## Abbreviations

NMPA: National Medical Products Administration
R&D: Research and development
TCM: Traditional Chinese medicine
DHP: Dihydropyridine
ARB: Angiotensin receptor blocker
DPP-4: Dipeptidyl peptidase-4
SGLT2: Sodium-dependent glucose transporters 2
NPC1L1: Niemann-Pick C1Like1
PCSK9: Proprotein Convertase Subtilisin/Kexin Type 9
ARNI: Angiotensin receptor-neprilysin inhibitor
GPR40: G protein-coupled receptor 40
IAPP: Islet amyloid polypeptide
PI: Principal investigators
MACCE: Major adverse cardiac and cerebrovascular events
CVD: Cardiovascular disease
ACEI: Angiotensin-converting enzyme inhibitors
ASCVD: Atherosclerotic coronary artery disease
AI: Artificial intelligence
hiPSC-CM: human induced pluripotent stem cell-derived cardiomyocyte.
