# Supplementary material for: The changing landscape of drug clinical trials on cardiometabolic diseases in China, 2009–2021
Source: Diabetol Metab Syndr. 2023 Apr 1;15:66. doi: 10.1186/s13098-023-01043-8 (PMC10067219; doi:10.1186/s13098-023-01043-8)

**Supplementary Material**

**Search Terms in the ‘Methods’ section**

**Coronary heart disease (CHD):** coronary heart disease, coronary atherosclerotic heart disease, coronary atherosclerosis, coronary artery, coronary syndrome, myocardial ischemia, myocardial infarction, angina pectoris, angina, chest pain.

**Stroke:** stroke, encephalorrhagia, subarachnoid hemorrhage, cerebral infarction, cerebral ischemia.

**Type 2 diabetes:** type 2 diabetes, diabetes Mellitus, diabetes, hyperinsulinemia, insulin resistance.

**Hypertension:** hypertension, secondary hypertension, hyperhomocysteinemia.

**Dyslipidemia:** dyslipidemia, hyperlipidemia, hypercholesterolemia, hypertriglyceridemia, combined hyperlipidemia , high density lipoprotein cholesterol, low density lipoprotein cholesterol.

**Obesity:** obesity, overweight.

**Supplemental Table 1: Distribution of clinical trials by drug types and mechanism in China**

| **Drug Classification** | **Number of Clinical Trails** |
| --- | --- |
| Thiazolidinediones | 19 |
| Glucokinase activator | 18 |
| If ion channel blocker | 15 |
| Omega-3 fatty acid | 11 |
| Thrombolytic Drugs | 10 |
| Loop diuretics | 9 |
| Aldosterone receptor antagonist | 8 |
| PPAR agonists | 8 |
| ARNI | 7 |
| Calcium channel blocker | 6 |
| PPARα/γ agonists | 6 |
| Folic acid | 6 |
| Cerebral vasodilator | 5 |
| GPR40 agonist | 5 |
| New oral hypoglycemic drugs | 5 |
| Niacin | 5 |
| Non dihydropyridine calcium antagonists | 5 |
| Renin inhibitor | 5 |
| Oral anti-angina agent | 5 |
| Potassium-sparing diuretics | 4 |
| Dopamine receptor agonists | 4 |
| IAPP analog | 4 |
| Intestinal lipase inhibitor | 4 |
| Angiogenic polypeptide | 3 |
| Hepatocyte growth factor | 3 |
| CETP inhibitor | 3 |
| Endothelin receptor antagonist | 2 |
| Histamine | 2 |
| ROMK inhibitors | 2 |
| Mesenchymal stem cell | 2 |
| Myocardial viability imaging agent | 2 |
| Farnesoid X receptor antagonist | 2 |
| Novel lipid-lowing small compound | 2 |
| Antisense oligonucleotides | 2 |
| Dopamine receptor agonist | 2 |
| Platelet activating factor antagonist | 2 |
| Selective calcium entry blocker | 2 |
| Adrenergic uptake inhibitor | 1 |
| ATP sensitive potassium channel opener | 1 |
| Adenosine A2A receptor agonist | 1 |
| Anti-IL-1β mono antibody | 1 |
| Prostaglandin receptor agonist | 1 |
| Thrombin inhibitor | 1 |
| ANGPTL3 mRNA antisense oligonucleotide | 1 |
| HMG-CoA reductase inhibitor | 1 |
| Lp (a) inhibitor | 1 |
| Amino acid | 1 |
| Antiplatelet agents | 1 |
| Apolipoprotein E mimetic peptide | 1 |
| Phosphodiesterase inhibitor | 1 |
| Serotonin 5-HT(2C) receptor agonist | 1 |
| Not available | 21 |

Abbreviations: PPAR, peroxisome proliferation activated receptors; ARNI, angiotensin receptor-neprilysin inhibitor; GRP40, G protein coupled receptor 40; IAPP, islet amyloid polypeptide; CETP, cholesterol ester transfer protein; ROMK, renal outer medullary potassium; IL-1β, Interleukin-1β; ANGPTL, angiopoietin-like 3 protein; HMG-CoA, Hydroxymethylglutaryl coenzyme A.

**Supplemental Figure 1. Distribution of phase and status for clinical trials for cardiometabolic diseases.**


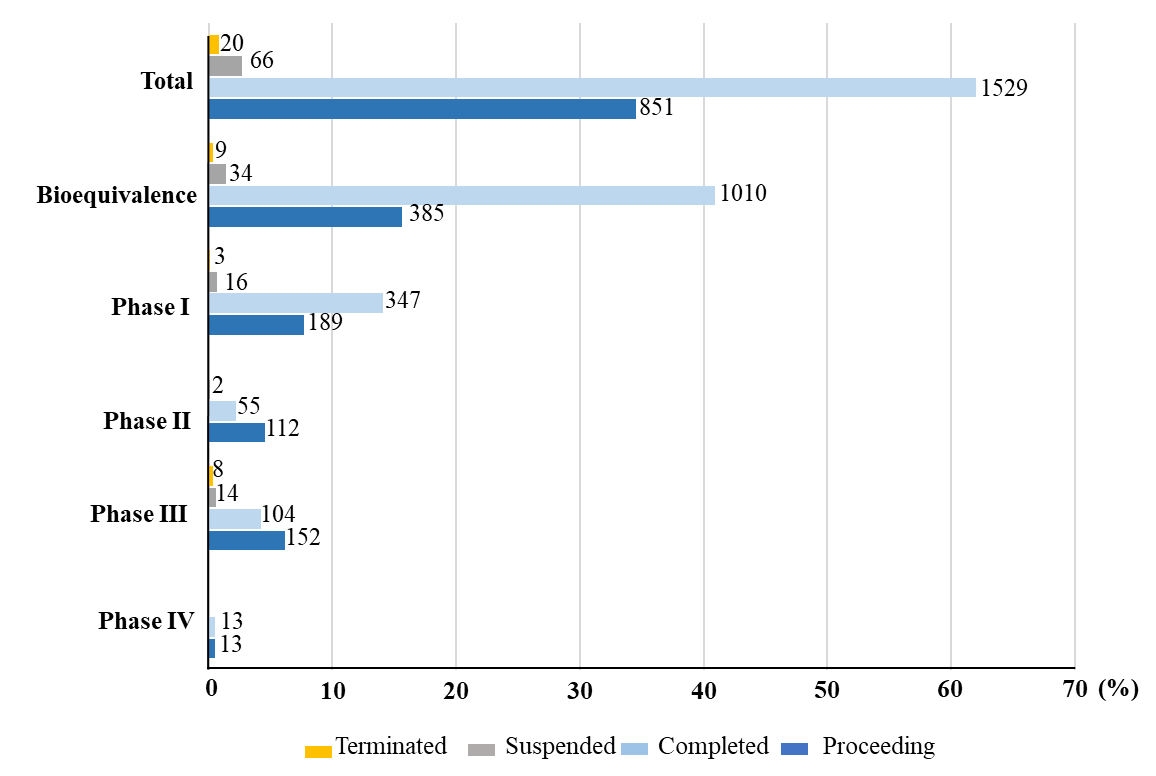


Numbers beside each column of the chart represent the actual number of trials.

**Supplemental Figure 2: Geographical Distribution of leading Units in China according to indications.**


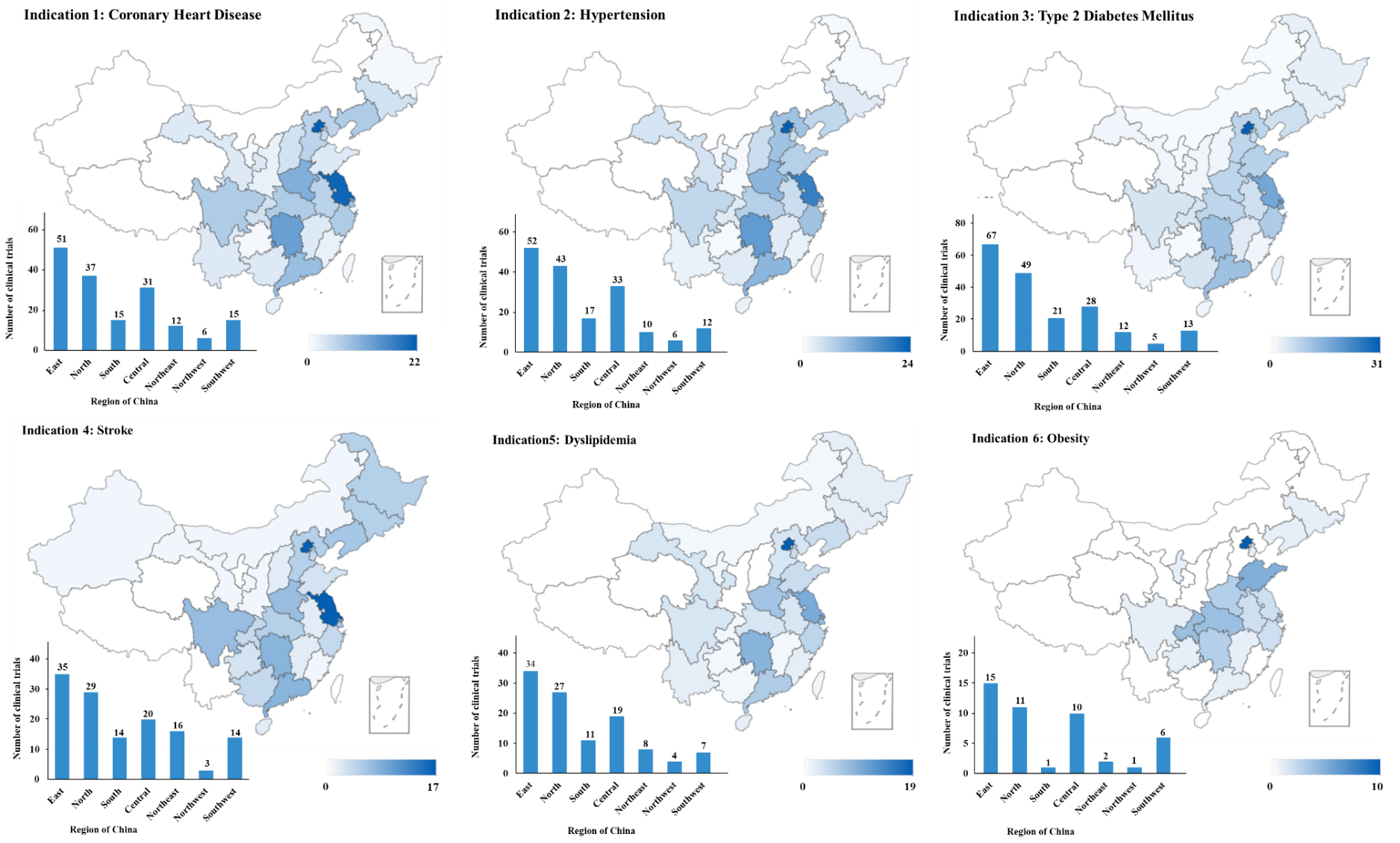

Supplement: Supplementary file 1 — Supplementary Material 1 [file 13098_2023_1043_MOESM1_ESM.docx]
